# Development and validation of cognitive ageing risk score (CARS) for early detection of subtle cognitive deficits in older people

**DOI:** 10.1186/s12877-024-04879-5

**Published:** 2024-03-21

**Authors:** Ada Wai Tung Fung, Allen Ting Chun Lee, Suk Ling Ma, Linda Chiu Wa Lam

**Affiliations:** 1 Department of Sport, Physical Education and Health, The Hong Kong Baptist University, Academic and Administrative Building, AAB931 Kowloon Tong, Hong Kong SAR China; 2 Department of Psychiatry, The Chinese University of Hong Kong, Sha Tin, Hong Kong SAR China

**Keywords:** Dementia risk, Early cognitive deficits, Vascular risks, Psychosocial risks, Lifestyle intervention, Alzheimer’s disease

## Abstract

**Background:**

Early cognitive deficits commonly seen in older people have not been well defined and managed in primary care. The objectives are (1) to develop and validate a new risk score to estimate the risk of dementia in Chinese older population; and (2) to evaluate the use of risk score in conjunction with cognitive screening in detecting early cognitive deficits in community older people.

**Methods:**

A development cohort of 306 cognitive healthy older adults aged 60 or above were followed for 6 years. A CARS was constructed using the estimated coefficients of risk factors associated with dementia at follow up. Validation was carried out in another five-year cohort of 383 older adults. The usefulness of CARS in detecting early cognitive deficits was evaluated.

**Results:**

Risk factors include older age, male gender, low level of education, poorly controlled diabetes, prolonged sleep latency, fewer mind body or light exercise, loneliness, and being apolipoprotein e4 carriers. A cutoff of CARS at -1.3 had a sensitivity of 83.9% and a specificity of 75.4% to predict dementia. The area under curve was 82.5% in the development cohort. Early cognitive deficits were characterized by impaired retention (*p* <.001, 95% CI 0.2–0.9) and attention (*p* =.012, 95% CI 0.1–0.8).

**Conclusion:**

The CARS can be used as a standard risk assessment of dementia or in conjunction with a computerized cognitive screening to evaluate a full cognitive profile for detecting early cognitive deficits. The result put forward the integration of risk algorithm into smart healthcare system to provide personalized lifestyle interventions.

## Background

Early detection of dementia is challenging due to much variance existed in the clinical diagnostic criteria. Reversion from mild cognitive impairment (MCI) to normal and accelerated progression from normal to dementia can be as high as 16% and 30% [1, 2]. A significant research priority has been focused on the use of blood-based screening and neuroimaging technique to verify the diagnosis of dementia in individuals with signs of cognitive decline or suspicion of dementia [3]. However, there is a long debate on how to preselect high risk individuals who may benefit from next level diagnostic workup or early management of cognitive decline.

The Lancet Commission in 2020 has reviewed and updated evidence to confirm twelve modifiable risk factors of dementia at different stages of life, suggesting an ongoing and cumulative risks of dementia can be attributable to an interplay of genetic, biological, psychosocial, and behavioral factors prior to dementia onset [4]. Many dementia risk scores have been developed to estimate the risk of dementia based on mid-life vascular risks or conversion from MCI to dementia in Western populations [5–10]. However, these prediction models may be less favorable to older Chinese adults whom lifestyle and characteristics are different. Psychosocial factors are also not well addressed in previously developed models. Furthermore, these models usually attribute points based on categorical scoring system, which may underestimate the probability of dementia with a given set of risk factors experienced by different individuals in the ageing path. Most importantly, many of these works are neither widely adopted in clinical practice nor well recognized by the public [11]. The rising need for an early risk tool in the increasingly health-conscious older populations to distinguish atypical cognitive deficits from age-related decline has not yet been met.

We proposed that dementia risk assessment can be used alone as an initial risk assessment of dementia or in conjunction with cognitive screening to provide a cognitive profile for detecting early cognitive deficits. The result could not only facilitate identification of high-risk targets, but also change the primary care practice for suggesting clinical workup from diagnostic testing to personalized lifestyle intervention for dementia prevention. The primary objective of this study was to develop and validate a cognitive ageing risk score (CARS) to estimate the risk of dementia based on a cluster of dementia-specific risk factors emerged during normal cognitive ageing in an older Chinese cohort. The secondary objective was to evaluate the use of risk scores in conjunction with cognitive screening in detecting early cognitive deficits in community older people. We hypothesized that CARS could substantiate cognitive screening to reveal a full cognitive profile that differentiates early cognitive deficits from normal cognitive ageing.

## Methods

### CARS construction in the development cohort

The development cohort was drawn from a dataset of a local community study based on 613 community older people interview between March 2012 and November 2013. Details of recruitment have been reported elsewhere [12]. A subgroup of 306 cognitively normal older participants at baseline were selected and reassessed on lifestyle activity pattern, medical morbidities, mental status, loneliness, social network, cognitive functioning, and sociodemographic information at an interval of 6.1 ± 0.6 years [13]. Of which, 5.6% (*N* = 17) were excluded from analysis due to missing baseline data (*N* = 3) and dementia as defined by the z-scores in a previous local epidemiological study [14]. As such, a total of 289 cases remained eligible for analysis. At 6-years follow up, 24.9% (*N* = 72) of the sample has converted to dementia. Baseline profile difference between converters and non-converters at 6-years was used to develop and construct the CARS. Inclusion of risk factors was based on association with neurodegeneration as suggested in literature. Ethical approval has been obtained from the research ethics committee of the Chinese University of Hong Kong.

### Validation of CARS

Validation of CARS was carried out in a cohort not involved in the risk score construction [15]. The validation cohort consisted of 383 non-demented cases with a mean follow up interval of 5.4 ± 0.3 years. The mean age was 69.9 ± 6.4 with mean years of education 6.4 ± 4.8. Half of the cohort (50.1%) was males. At follow up, 18.3% (*N* = 70) of the sample has declined. Performance of CARS and association with dementia at follow up were evaluated based on ROC.

### Dementia diagnosis

The diagnosis of dementia was defined based on the cognitive z-scores criteria of a demented sample in a previous local epidemiological study using the same set of cognitive assessments [14]. Cognitive performance was assessed using Cantonese version of the mini-mental state examination (CMMSE), 10-word list learning and ten-minute delayed recall tests, category verbal fluency test (CVFT), and digit span and visual span tests. A z-score was computed for each neurocognitive test with reference to the age and educational matched control mean and standard deviation (SD) at baseline. These cognitive measures demonstrated satisfactory performance in predicting dementia with 91.5% sensitivity and 62% specificity in a local five-year follow-up community survey [15].

### Risk assessments

Vascular risks include the presence of hypertension, diabetes mellitus or hyperlipidemia, obesity, smoking, or drinking. Hypertension was defined by self-report of a clinical diagnosis; or a measurement of a systolic blood pressure ≥ 130 mm Hg and/or diastolic blood pressure ≥ 80 mm Hg; or on antihypertensive medication [2]. diabetes mellitus was defined by self-report of a clinical diagnosis; or having a fasting plasma glucose level of ≥ 7 mmol/L; or on blood glucose lowering medication; [3] hyperlipidemia was defined by self-report of a clinical diagnosis; or having a total cholesterol level of > 8 mmol/L or low-density lipoprotein (LDL) level of < 4.9 mmol/L, or on lipid lowering medication. Severity of each vascular disease followed the rating system of Cumulative Illness Rating Scale (CIRS) from 0 to 4 as follows: 0 = No impairment to organs/system; 1 = Mild impairment (No interference, normal activity; treatment required; prognosis excellent); 2 = Moderate impairment (Interference, normal activity; treatment required; prognosis good); 3 = Severe impairment (Disability; urgent treatment required; prognosis guarded); and 4 = Extremely severe impairment (Life threatening; treatment is emergent or of no avail; grave prognosis) [16]. Higher score indicates the increasing severity of impairment and urgency of medical intervention. Overweight was defined as having a Body Mass Index (BMI) larger than 30 kg/m^2^. Smoking status was categorized as not smoking or currently smoking with number of cigarettes smoking per day. Alcohol consumption was categorized as never or drinking.

Psychological risks include mental health status and loneliness. Mental health status was assessed using the Revised Clinical Interview Schedule (CIS-R) [17]. It focuses on changes in neuropsychiatric symptoms and evaluates potential mood and behavioral changes. The total score ranges from 0 to 57. A higher score indicates higher levels of symptom severity. The cut-off score of 12 or above for significant risk of mental illness has been validated. It generates a diagnosis of common mental disorders, according to the Tenth Revision of the International Classification of Diseases of the World Health Organization diagnostic criteria (ICD-10). Level of loneliness was assessed by the validated Chinese version of the 6-item De Jong Gierverg’s Loneliness Scale [18]. The overall loneliness score ranges from 0 to 6, with a higher score indicating a higher level of loneliness. A cut-off of 2 or more indicates experiencing loneliness.

Behavioral risks include level of physical exercise and sleep quality. Physical exercise was divided into light exercise, mind body (MB) exercise, and strenuous exercise [19]. Light exercise includes walking alone, walking the pet, muscle stretching, and other forms of toning exercises. MB exercise encompasses Six forms, Tai chi, Qi gong, Pak Duen Kam, yoga, and dancing. Strenuous exercise includes moderate to vigorous intensity exercises, such as jogging, hiking, swimming, ball games, etc. Level of participation was evaluated based on number of types, frequency (daily, several times a week, weekly, several times a month, monthly, bimonthly and 3 months or longer), and duration in minutes during the past 12 months. The Chinese version of the Pittsburgh sleep quality index (CPSQI) assesses sleep quality, including sleep duration and latency, the frequency and severity of specific sleep problems [20]. It consisted of 19 self-rated questions and five questions rated by their bed partner. The scale has a range of scores from 0 to 21, a higher score indicates worse sleep quality. The cut-off score for poor sleeper is 6.

Genetic risk was measured by the presence of apolipoprotein e4 (ApoE4), so participants with one or more copies were categorized as e4 carriers.

### Major covariates

Sociodemographic variables including age, years of education, and gender were obtained. Medical morbidity will be measured by the non-vascular components of the Cumulative Illness Rating Scale (CIRS). It assesses the clinical illness burden of medical diseases in the following categories: (1) cardiac disease; (2) respiratory diseases; (3) eye, ear, nose, throat (EENT) diseases; (4) upper gastrointestinal tract diseases; (5) lower gastrointestinal tract diseases; (6) hepatic diseases; (7) renal diseases; (8) other genitourinary diseases; (9) musculoskeletal and Integumentary diseases; (10) neurologic diseases; (11) psychiatric diseases. Each item is rated along a continuum from 0 to 4. The total score is the sum of all bodily system scores ranging from 0 to 40.

### Data analyses

#### CARS construction

To generate a predictive model, all cases in the development cohort with detectable cognitive impairments were excluded, leaving a cognitively normal group for dementia-specific risk analysis. Independent sample t-test or Chi-square test was used to compare the baseline profile difference between converters and non-converters at 6-years in the development cohort. Association with incident dementia attributable to deviations in each biopsychosocial parameter from baseline was examined using logistic regression. Factors with significant associations were put in a single regression model to obtain the estimated regression coefficients. Accuracy and cut-off of the CARS was determined by the area under the receiver operating characteristic curve (AUC).

The following formula was used to calculate the CARS for the development and validation cohort:

$$ \begin{aligned} {\text{CARS}} & ={\text{Intercept}}+({\text{B}}_{\text{Age}}*{\text{Age}}) + ({\text{B}}_{\text{Edu}}*{\text{Education}}) \\ &\quad + ({\text{B}}_{{\text{Risk}}1\ldots {\text{RiskN}}}*{\text{Risk}}1\ldots {\text{RiskN}}) \end{aligned} $$

The risk of dementia was estimated using the following formula:

$$ {\text{P}}({\text{Dementia}}) = \frac{\text{exp}\left(\text{C}\text{A}\text{R}\text{S}\right)}{1+\text{exp}\left(\text{C}\text{A}\text{R}\text{S}\right)}$$

#### Implementation of CARS

To evaluate the use of risk score in conjunction with cognitive screening in detecting early cognitive deficits, we extracted a subgroup of 286 older people who has completed the computerized Hong Kong– Vigilance and Memory Test (HK-VMT) in the development cohort [21]. The HK-VMT classification was stratified by CARS cutoff to form four groups, namely cognitively normal (CN), cognitively normal with high-risk (CH), mild cognitive impairment with low risks (MCI-L), and MCI with high risks (MCI-H). The cognitive profile of HK-VMT among all groups were then examined and compared based on four cognitive domains on retrieval, retention, attention, and visuospatial memory using ANOVA test. Magnitude of retrieval was measured by subtracting the delayed recall from the average immediate recall. The difference was then converted into z-score by comparing to the age- and education-matched norms at baseline. A positive z-score indicates intact ability when compared to matched norm, while a negative value indicates impairment. Magnitude of retention was measured by averaging the three acquisition trials in the word list learning test. Magnitude of attention and visuospatial memory was measured by averaging the sum of all trials. Similarly, all raw scores were converted to z-score compared to the matched norm at baseline. Group differences for HK-VMT measures were established based upon one-way ANOVA. Data analyses were performed in IBM SPSS Statistics 28.0 for windows. The statistical significance of all analyses was set as *p* <.05.

## Results

### Characteristics of the development cohort (*N* = 289)

Converters were significantly older, less educated, being male, being smokers, having diabetes, feeling lonely, poor sleeper, fewer mind body exercise (≥ 15 min), fewer light exercise (≥ 45 min), poorer cognitive functioning, and being ApoE4 carriers. Table 1 displayed the baseline profile of the participants who had and had not converted to dementia at six years follow up.

**Table 1:** Comparison of baseline demographic and cognitive profile of older adults by dementia conversion at 6 years

	All (*N* = 289)		Stable (*N* = 217)		Converter (*N* = 72)		T-test/r
	Mean	S.D.	Mean	S.D.	Mean	S.D.	p-value
Age	67.8	5.9	66.7	5.4	71.3	6.0	< 0.001
Years of Education	10.3	4.8	10.9	4.6	8.4	5.0	< 0.001
Male (%)	44.4	-	40.6	-	56.9	-	0.015
Smoking (%)	6.5	-	4.6	-	12.5	-	0.019
Drinking (%)	25.2	-	24.9	-	23.6	-	0.828
BMI	23.5	3.5	23.7	3.7	23.0	2.5	0.099
CIRS Total	2.7	1.7	2.6	1.6	3.0	2.0	0.133
Severity of diabetes	0.4	1.0	0.3	0.8	0.6	1.3	0.008
Severity of hypertension	0.5	0.5	0.4	0.5	0.5	0.5	0.336
Severity of hyperlipidaemia	0.4	0.5	0.4	0.5	0.4	0.5	0.913
CISR total	3.3	5.7	2.9	5.4	4.3	6.6	0.074
Loneliness total	2.2	1.7	2.0	1.7	2.5	1.6	0.025
Lonely (%)	62.0	-	73.6	-	58.1	-	0.019
**CPSQI total**	5.6	3.4	5.3	3.4	6.4	3.6	0.024
Sleep latency	0.9	1.0	0.8	0.9	1.2	1.1	0.003
Sleep Efficiency	0.8	1.1	0.7	1.0	1.1	1.2	0.011
**No. of physical exercises**							
Light (≥ 15 min/wk)	0.8	0.7	0.8	0.7	0.9	0.8	0.285
Light (≥ 30 min/wk)	0.7	0.6	0.7	0.6	0.7	0.7	0.800
Light (≥ 45 min/wk)	0.4	0.6	0.5	0.6	0.3	0.5	0.007
Light (≥ 60 min/wk)	0.4	0.5	0.4	0.5	0.3	0.5	0.026
MB (≥ 15 min/wk)	0.6	0.9	0.6	0.9	0.3	0.6	0.001
MB (≥ 30 min/wk)	0.5	0.8	0.5	0.8	0.3	0.6	0.007
MB (≥ 45 min/wk)	0.4	0.7	0.4	0.7	0.2	0.6	0.022
MB (≥ 60 min/wk)	0.4	0.6	0.4	0.7	0.2	0.6	0.024
Strenuous (≥ 15 min/wk)	0.5	0.8	0.6	0.9	0.6	0.7	0.398
Strenuous (≥ 30 min/wk)	0.5	0.8	0.5	0.8	0.5	0.7	0.909
Strenuous (≥ 45 min/wk)	0.4	0.7	0.4	0.7	0.3	0.6	0.297
Strenuous (≥ 60 min/wk)	0.4	0.7	0.4	0.7	0.3	0.6	0.244
CMMSE	28.9	1.2	29.0	1.1	28.6	1.2	0.003
CVFT	47.2	9.0	48.8	8.6	42.5	8.5	< 0.001
Delayed Recall	7.1	1.6	7.4	1.5	6.1	1.5	< 0.001
ApoE4 carrier (%)	18.0	-	15.1	-	30	-	0.010

BMI = Body Mass Index; CIRS = the Cumulative Illness Rating Scale; CISR = Revised Clinical Interview Schedule; CPSQI = Chinese version of the Pittsburgh sleep quality index; MB = Mind body; CMMSE = Cantonese version of the mini-mental state examination; CVFT = Category verbal fluency test; ApoE4 = Apolipoprotein e4. S.D.= Standard deviation

### Association between biopsychosocial risks and converters

Biopsychosocial parameters that differed significantly at baseline between groups were examined individually with the association with incident dementia at 6 years. Univariate analysis demonstrated significant association in age (B = 0.1, Exp(B) = 1.1, 95% C.I. 1.1–1.2, *p* <.001), years of education (B=-0.1, Exp(B) = 0.9, 95% C.I. 0.8–1.0, *p* <.001), being males (B = 0.7, Exp(B) = 1.9, 95% C.I. 1.1–3.3, *p* =.016), severity of diabetes (B = 0.4, Exp(B) = 1.4, 95% C.I. 1.1–1.8, *p* =.003), smoking (B = 1.1, Exp(B) = 3.0, 95% C.I. 1.2–7.6, *p* =.024), prolonged sleep latency (B = 0.4, Exp(B) = 1.5, 95% C.I. 1.1–2.0, *p* =.004), sleep efficiency (B = 0.4, Exp(B) = 1.4, 95% C.I. 1.1–1.8, *p* =.005), being lonely (B = 0.7, Exp(B) = 2.0, 95% C.I. 1.1–3.6, *p* =.020), mind body exercise (≥ 15 min) (B=-0.6, Exp(B) = 0.6, 95% C.I. 0.4–0.9, *p* =.007), light exercise (B=-0.7, Exp(B) = 0.5, 95% C.I. 0.3–0.9, *p* =.015), and being ApoE4 carrier (B = 0.9, Exp(B) = 2.4, 95% C.I. 1.2–4.8, *p* =.012).

### Development of CARS

Two models have been explored and formulated based on statistically significant predictors through stepwise selection. The first one includes vascular, psychosocial, and behavioral risk factors. The second one includes APOE ε4 status. The CARS was further categorized using cut-off guided by the receiver-operator characteristic (ROC) curve and observed relationship with incident dementia at 6-year. Table 2 demonstrates the association with incident dementia attributable to identified biopsychosocial parameters from baseline.

**Table 2:** Association with identified biopsychosocial parameters with incident dementia at 6 years using logistic regression

Risk factors	Model 1				Model 2			
	B	Exp(B)	95% C.I.	Sig.	B	Exp(B)	95% C.I.	Sig.
Age	0.1	1.1	1.1–1.2	< 0.001	0.1	1.1	1.1–1.2	< 0.001
Years of education	−0.1	0.9	0.8-1.0	0.010	−-0.1	0.9	0.8-1.0	0.003
Male	1.0	2.8	1.3–5.9	0.006	1.2	3.3	1.4–7.7	0.007
Severity of diabetes	0.3	1.4	1.0-1.9	0.026	0.4	1.4	1.0–2.0	0.037
Sleep latency	0.5	1.7	1.2–2.3	0.004	0.6	1.7	1.2–2.6	0.007
Being lonely	1.1	2.9	1.4–6.1	0.004	1.3	3.5	1.5–8.4	0.005
MB (≥ 15 min/wk)	−0.6	0.5	0.3–0.9	0.015	−0.5	0.6	0.3-1.0	0.044
Light exercise (≥ 45 min/wk)	−1.1	0.3	0.2–0.7	0.003	−1.5	0.2	0.1–0.5	0.001
ApoE4 carrier	−	−	−	−	1.4	3.9	1.6–9.4	0.003
Intercept	−9.1	−	−	−	−10.2	−	−	−

MB = Mind Body Exercise; ApoE4 = Apolipoprotein e4

### Model 1 includes vascular, psychological and lifestyle risk factors. Model 2 also includes APOE ε4 status

In model 1, the CARS has a mean of -1.6 ± 1.6. The mean CARS of stable participants and converters were– 2.0 ± 1.4 and − 0.2 ± 1.3 (t=-9.5, *p* <.001) respectively. The area under curve (AUC) is 82.5% (95% CI 0.8–0.9, *p* <.001). A cutoff at -1.3 or above was chosen with a sensitivity and specificity of 83.9% and 75.4%. A total of 43.1% of the population is above cut-off, indicating a higher level of risk. The CARS has a significant association with incident dementia at 6 years (B = 1.0, Exp(B) = 2.7, 95% CI 2.1–3.6, *p* <.001). Based on the CARS cutoff, the probability of dementia in 6 years in high risk older people is 46.0%.

In model 2, the mean CARS was − 1.8 ± 2.0. The mean CARS of stable participants and converters were − 2.5 +/- 1.7 and 0.04 ± 1.5 (t=-9.7, *p* <.001). The AUC is 86.5% (95% CI 0.8–0.9, *p* <.001). A cutoff at -1.5 or above has a sensitivity and specificity of 83.9% and 74.9%. A total of 41.6% of the participants are above cut-off, indicating a higher level of risk. The CARS has a significant association with incident dementia at 6 years (B = 1.0, Exp(B) = 2.7, 95% CI 2.0–3.6, *p* <.001). Based on the CARS cutoff, the probability of dementia in 6 years in high risk older people is 51.3%.

### Validation of CARS

The mean CARS in the validation cohort was − 1.1 ± 1.4. The mean CARS of stable participants and converters were − 1.4 ± 1.4 and − 0.4 ± 1.6 (t=-3.7, *p* <.001). The AUC is 71.1% (95% CI 0.6–0.8, *p* <.001). A total of 46.6% of the participants were above the CARS cutoff. The CARS has a significant association with incident dementia at 5 years (B = 0.4, Exp(B) = 1.5, 95% CI 1.2– 2.2, *p* =.002). Based on the CARS cutoff in model 1, the probability of dementia in 5 years in high risk older people is 40.4%.

### Cognitive and Risk Profiles by CARS in conjunction with HK-VMT

The use of CARS in conjunction with cognitive screening in detecting early cognitive deficits has been evaluated in a subgroup of 286 older people who completed the HK-VMT in the development cohort. By HK-VMT classification, 212 (74.1%) have normal cognition and 74 (25.9%) have MCI. The CARS cutoff was then applied to the HK-VMT classification, 49.3% (*N* = 141) was cognitively normal (CN), 24.8% (*N* = 71) was cognitively normal with high-risk (CH), 8.4% (*N* = 24) had MCI with low risks (MCI-L), and 17.5% (*N* = 50) had MCI with high risks (MCI-H).

A one-way ANOVA was conducted to compare the cognitive profiles of HK-VMT by risk stratification (CN, CH, MCI-L, and MCI-H). The results showed that significant differences in the HK-VMT total score (F = 107.2, *p* <.001, η2 *p* =.533), retrieval (F = 17.4, *p* <.001, η2 *p* =.156), retention (F = 49.0, *p* <.001, η2 *p* =.343), attention (F = 14.4, *p* <.001, η2 *p* =.133), and visuospatial memory (F = 17.6, *p* <.001, η2 *p* =.158), based on the cognitive status stratified by CARS. The HK-VMT measures of MCI-H were the lowest of all groups. A post hoc analysis showed significant differences between CN and CH in the total score (*p* <.001, 95% CI 0.1–0.7), retention (p = < 0.001, 95% CI 0.2–0.9), and attention (*p* =.012, 95% CI 0.1–0.8). There were significant differences between CH and MCI-L in total score (*p* <.001, 95% CI 0.7–1.6) and visuospatial memory (*p* =.006, 95% CI 0.2–1.6). There were significant differences between MCI-L and MCI-H in retrieval (*p* =.010, 95% CI 0.1–1.4) and retention (*p* =.042, 95% CI 0.01–1.1). Table 3 showed the ANOVA and post hoc analysis for comparisons between groups according to the HK-VMT measures. Logistic regression was performed to explore the association between cognitive status and dementia at 6 years. Relative to CN, the likelihood in having dementia in CH was 9.4 (95% CI: 4.2–21.1, *p* <.001), and 13.9 in MCI-H (95% CI: 5.8–33.3, *p* <.001), but MCI-L was not associated with dementia at 6 years (*p* =.125).

**Table 3:** ANOVA for comparisons between groups according to Hong Kong-Vigilance and Memory Test (HK-VMT) measures

			Cognitive states by CARS															
	All (*N* = 286)		CN^1^ (*N* = 144)		CH^2^ (*N* = 68)		MCI-L^3^ (*N* = 24)		MCI-H^4^ (*N* = 50)		Sig.	Post hoc comparison			Effect size*			
	M	(SD)	M	(SD)	M	(SD)	M	(SD)	M	(SD)		1 vs. 2	2 vs. 3	3 vs. 4				
Total	0.05	1.05	0.62	0.74	0.22	0.79	−0.94	0.52	−1.33	0.62	< 0.001	0.001	< 0.001	-		0.533§		
Retrieval	0.00	1.06	0.28	0.93	0.10	1.07	−0.09	0.81	-0.87	1.08	< 0.001	−	−	0.009		0.156§		
Retention	0.07	0.97	0.55	0.87	0.00	0.76	−0.43	0.59	−0.96	0.69	< 0.001	< 0.001	−	0.035		0.343§		
Attention	0.04	0.96	0.35	0.81	−0.06	0.93	−0.16	0.99	−0.59	1.04	< 0.001	0.011	−	−		0.133§		
VM	0.12	1.19	0.49	1.19	0.18	1.11	−0.69	0.83	−0.63	0.93	< 0.001	−	0.005	−		0.158§		

CARS: Cognitive Ageing Risk Score, VM: Visuospatial Memory; CN: Cognitively Normal; CH: cognitively normal with high-risk; MCI-L: Mild Cognitive Impairment with low risks; MCI-H: MCI with high risks; M: Mean; SD: Standard Deviation

*Note* * Partial eta-squared (η²) was used to compute effect size: † small ≥ 0.01, ‡ medium ≥ 0.06, § large ≥ 0.14

## Discussion

The purpose of our study is to develop a dementia risk model that could characterize early cognitive deficits in high risk or asymptomatic older people. Our study identifies several key risk factors that predispose the Chinese older population to dementia. The dementia-associated risk factors include old age, low level of education, male gender, poorly controlled diabetes, infrequent light or mind body exercise, prolonged sleep latency, loneliness, and being ApoE4 carriers. They are consistent with and well supported by previous local longitudinal studies on dementia risk [13, 22, 23]. The CARS was composed as the sum of these robust risk factors to provide prognostic value to suggest personalized interventions and risk surveillance based on person-centered cognitive outcomes.

Compared to previous works on risk scores construction, our approach may be more conservative in term of risk selection. Previous models have included younger sample or factors that are not associated with dementia in their own study, which may overstate dementia risk in their sample [5–10]. However, our risk score is more precise than other risk models. We have selected a comprehensive list of well-established biopsychosocial risk factors in the Chinese older populations and included only those associated with dementia in our sample. This provides a strong foundation in favour of screening of lifestyle risk as part of the neurocognitive evaluation for older people. We have included loneliness in the risk assessment model to emphasize the importance of managing mental health risk as much as treating the well-established vascular risks. In addition, the dementia risk can be adjusted through increasing light or mind body exercise, which is an age and culturally appropriate recommendation for cognitive enhancement in older people. Our risk model could contribute to a more unified management from mental health and physical health simultaneously.

To our knowledge, this is the first time to implement risk score into computerized cognitive evaluation in primary setting. The CARS was first constructed and externally validated using two longitudinal cohorts of healthy older people without significant cognitive impairment. The AUC only improves slightly by including ApoE4 in the model, indicating that the CARS has adequate accuracy to be used as an initial standard risk assessment of dementia to identify high risk older adults in primary care setting. Furthermore, the utilization of CARS in conjunction with cognitive screening has refined the current classification of cognitive function in community older people. It provides a full cognitive profile that differentiates early cognitive deficits and MCI from normal older adults. Therefore, the combined assessment is a valuable tool to categorize cognitive impairments more precisely and to inform older people of their tendency in progressive decline along the continuum of cognitive ageing.

Interestingly, our result indicates that poor attention and retention could be an early sign of cognitive decline indicating the need of early intervention, while poor visuospatial skills could distinguish older people with normal cognition from MCI. This result is consistent with other studies where decreasing attention, inability to learn, and poor visuospatial skills are observed in older people with subjective cognitive decline [24]. These findings may be associated to the brain pathology in the prefrontal cortex and medial temporal regions that are sensitive to the progression of vascular conditions, such as diabetes [25]. Therefore, this result supports the use of risk stratification in conjunction with cognitive evaluation for identification of early target, especially in the subtle changes that are not easily detectable in routine clinical examination. It could help to detect probable dementia in otherwise undiagnosed cases in the community.

A strength of our study is that the dementia case ascertainment was based on the cognitive performance of a demented sample assessed by the same set of cognitive assessments, reducing the likelihood of diagnostic bias. In addition, our development cohort and validation cohort both have an observation period of at least 5 years and were conducted in a local community sample excluding all significant cognitive impairments at baseline, demonstrating the significance of our finding on early detection of preclinical dementia in Chinese older people. Most importantly, each factor has an unequal weight on cognitive decline. The use of regression coefficient enables us to explain individual differences in the susceptibility of developing dementia and provides a precise estimation of individual risk with a given set of dementia-specific risk factors.

This study has several limitations. First, misdiagnosis of dementia is possible due to the lacking biomarkers or neuroimaging findings to confirm the presence of pathology in converters at 6 years. Second, the generalizability of using the CARS to other cognitive evaluation remains unknown, because risk stratification was carried out based on the cognitive state detected by the computerized cognitive test. Therefore, the effect may be limited to the use of HK-VMT only. It will be important to replicate these findings using other cognitive assessments in the future.

The use of CARS in conjunction with HK-VMT has enlarged the detection net to include subtle cognitive deficits in asymptomatic individuals for very early care. The integrated platform will provide information on health and cognitive profile to facilitate discussion between healthcare professionals and their clients for early lifestyle modification. The CARS does not only facilitate detection of subtle cognitive changes before irreversible cognitive changes occur, but also provides insight to older adults who may not be aware of the dementia risk due to poorly managed health conditions or lifestyle. More importantly, CARS can be used to select personalize interventions base on dementia related risk. It may help to indicate and suggest a specific combination for individual to maximize the benefit of lifestyle modification, because CARS is calculated based on personal background, diabetes, level of physical activity, and loneliness. Changes in CARS may indicate the need to refine or strengthen specific training or intervention as cognitive function improved or declined over time. Therefore, CARS can facilitate continuous self-monitoring and self-management of dementia risk according to personal lifestyle preferences and care plan. Further study of the clinical utility and reliability of this refined classification will be needed. The predictability of CARS should be continually tested and improved with a larger and more representative cohort in the future.

## Conclusions

The use of CARS in conjunction with computerized cognitive screening can be useful for cognitive function classification and differentiation in preclinical dementia. The cognitive profiles identified based on the evaluation in attention and visuospatial memory are sensitive to subtle changes in cognition in normal older people. The refined classification may have clinical utility for suggesting further clinical workup from diagnostic testing to personalized lifestyle intervention or drug treatment in primary care practice. While the impact of early detection on dementia risk reduction is yet to be evaluated, information generated from the risk assessment can assist service providers or informal carers to closely monitor the cognitive health of older people and to suggest proper early care or activities. Our findings also put forward the integration of risk algorithm into smart monitoring and care systems that can customize management for the at-risk group before onset of dementia.

## Data Availability

The datasets generated and/or analyzed during the current study are not publicly available due to confidentiality agreements with research collaborators but are available from the corresponding author on reasonable request.
